# Applications and limitations of fitting of the operational model to determine relative efficacies of agonists

**DOI:** 10.1038/s41598-019-40993-w

**Published:** 2019-03-15

**Authors:** Jan Jakubík, Alena Randáková, Vladimír Rudajev, Pavel Zimčík, Esam E. El-Fakahany, Vladimír Doležal

**Affiliations:** 1 Institute of Physiology CAS, 142 20 Prague, Czech Republic; 2 Department of Experimental and Clinical Pharmacology, University of Minnesota College of Pharmacy, Minneapolis, MN 55455 USA

## Abstract

Proper determination of agonist efficacy is essential in the assessment of agonist selectivity and signalling bias. Agonist efficacy is a relative term that is dependent on the system in which it is measured, especially being dependent on receptor expression level. The operational model (OM) of functional receptor agonism is a useful means for the determination of agonist functional efficacy using the maximal response to agonist and ratio of agonist functional potency to its equilibrium dissociation constant (K_A_) at the active state of the receptor. However, the functional efficacy parameter τ is inter-dependent on two other parameters of OM; agonist’s K_A_ and the highest response that could be evoked in the system by any stimulus (E_MAX_). Thus, fitting of OM to functional response data is a tricky process. In this work we analyse pitfalls of fitting OM to experimental data and propose a rigorous fitting procedure where K_A_ and E_MAX_ are derived from half-efficient concentration of agonist and apparent maximal responses obtained from a series of functional response curves. Subsequently, OM with fixed K_A_ and E_MAX_ is fitted to functional response data to obtain τ. The procedure was verified at M_2_ and M_4_ muscarinic receptors fused with the G_15_ G-protein α-subunit. The procedure, however, is applicable to any receptor-effector system.

## Introduction

The term “efficacy” is linguistically defined as the ability to produce the desired or intended effect. In pharmacological terms, efficacy means the ability of a chemical to produce a functional response in a cell, tissue or organ. Absolute quantification of efficacy is impossible. Thus, efficacy is rather described in relative terms, e.g. an agonist that produces a more robust maximal response than another is considered more efficacious. In this case the latter agent is described as a partial agonist. However, mere comparison of the magnitude of the maximal response maybe misleading. This is because the apparent maximal response to an agonist is not only a function of its efficacy, but is also dependent on the level of expression of the receptor and signalling entities. For example, a partial agonist may produce an apparent maximal response equal to that of a full agonist in a system with high efficiency of coupling of the receptor to intracellular signal transduction pathways. The relationship between agonist concentration and proportion of receptor occupancy is a mere function of agonist affinity for the given receptor. However, a gradual increase in receptor expression results in proportionally higher numbers (rather than relative proportion) of receptor-agonist complexes. A maximum response is eventually attained due to saturation of downstream effector systems. As a consequence, a partial agonist may reach the maximal response as a full agonist in a high receptor expression system. The potency of an agonist is a measure of the concentration required for exerting a certain level of biological activity, e.g. concentration required to produce its half-maximal effect (EC_50_). Using the same argument as above, a smaller fraction of receptors in a high expression system is needed to form the same number of receptor-agonist complexes. Thus, a lower concentration of agonist is required to produce the same response (i.e. increased “apparent” potency) at higher levels of receptor expression. Taken together, both observed maximal response and potency are system-dependent.

A critical step in screening chemical libraries for new selective agonists is the proper determination of agonist activity that is system-independent. The same criterion is also needed to rule out that apparent agonist selectivity at a given receptor versus another is an artifact of the test system or assay. Based on these premises the operational model (OM) of pharmacological agonism was formulated^1,2^. This basic OM calculates a parameter τ termed “operational efficacy” of agonist from “objective” parameters, namely the equilibrium dissociation constant of agonist (K_A_) at the active state of the receptor and the maximal response of the system (E_MAX_). OM thus can also rank efficacies of agonists whose responses approach the system E_MAX_. Since its formulation, OM has been widely applied in pharmacological analysis in its original form as well as in various modifications and extensions^3,4^.

The major pitfall of application of the OM is that all 3 parameters (K_A_, E_MAX_ and τ) are inter-dependent. This constitutes possible difficulties and special requirements in the fitting of the OM to data. We have addressed these issues in the part of our study dedicated to theoretical analysis. We also analysed options to improve robustness of fitting of OM to experimental data. One established way to apply the OM is to determine the value of K_A_ experimentally. However, an agonist is not a “mere observer”. Rather, its interaction with the system components affects its affinity^5^. Another established way to fit the OM is to fit at least two dose-response curves sharing some parameters^6^. Finally, we propose a new two-step procedure for the fitting of the OM. We demonstrate benefits of the procedure on the system where, in binding experiments, agonist binding occurs solely to receptors in an inactive conformation, making it impossible to calculate K_A_ at the active state of the receptor. We also show that this procedure affords proper ranking of agonist efficacy by fitting of the OM, even when maximal responses to various agonists approach E_MAX_ of the system. It is impossible to perform direct fitting of OM to data under these conditions using conventional analysis.

## Results

### Evaluation of the theoretical operational model

**Figure 1:**
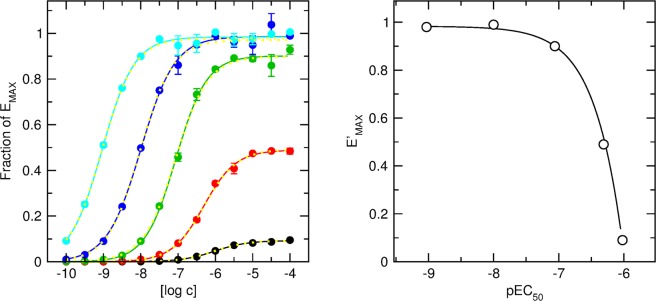
Fitting of operational model of agonism (OM) to theoretical concentration-response curves. Left: Simulated data for an agonist with log K_A_ = −6.0 in 5 systems with coupling efficacy τ varying from 0.1 (black circles) to 1000 (cyan circles). Concentration-response curves were fitted to the simulated data according to Eq. (1). Right: Resulting apparent maximal responses (E’_MAX_) were plotted against resulting half-efficient concentrations (EC_50_). Maximal possible response of the system (E_MAX_) and affinity of agonist for receptor (K_A_) were obtained by fitting Eq. (6). Calculated E_MAX_ and K_A_ were used in fitting OM Eq. (1) to the data in the left graph (yellow lines). Resulting τ are in Table 3.

The operational model of agonism (OM) is described by following equation:

1 $${\rm{Response}}=\frac{[A]\ast \tau \ast {E}_{{\rm{MAX}}}}{[A]\ast ({\rm{\tau }}+1)+{{\rm{K}}}_{A}}$$

where [A] is the concentration of an agonist, E_MAX_ is the maximal response of the system, K_A_ is the equilibrium dissociation constant of the agonist-receptor complex and τ is the operational factor of efficacy. As can be seen, all 3 parameters (E_MAX_, K_A_ and τ) are bound. Clearly, the asymptote of the response curve is given by a product of τ and E_MAX_. Thus, an increase in τ is associated with lowering E_MAX_ and vice versa. Similarly, the inflection point of the response curve is given by the ratio of K_A_ to τ; thus, higher values of τ are accompanied by a corresponding increase in K_A_, and vice versa. To evaluate the impact of parameter interdependence on non-linear regression, simulated data of theoretical agonists were generated (see Supplementary information) and the OM was fitted to the simulated data by various procedures. Simulated data of functional response to agonist with 1 μM affinity (K_A_) were generated by calculation of functional response according to the OM Eq. (1) at 13 concentrations ranging from 0.1 nM to 0.1 mM and addition of 3% of noise (Fig. 1, left). Five sets of simulated data were calculated for 5 values of τ, ranging from 0.1 (Fig. 1, black) to 1000 (Fig. 1, cyan) with 10-fold increase in each step. Fitting of the OM to individual datasets resulted in a large error margin of calculated parameters (Table 1). Calculated parameters were correct (close to the simulated ones) only when initial parameter estimates were set to simulated values. When initial parameter estimates were under- or overestimated the calculated parameters were wrong (different from simulated ones) (Supplementary information, Fig. SI1). Specifically, a low initial estimate of E_MAX_ resulted in overestimated τ and high initial estimate of E_MAX_ resulted in underestimated τ in most cases. This confirms that all 3 parameters of the OM are inter-bound. Under these conditions the error margin is so large that it is impossible to estimate OM parameters from individual fits.

**Table 1:** Fitting OM to simulated data.

Dataset	τ	E_MAX_	log K_A_
**Individual fits**			
A	0.1017 ± 143155	1.00 ± 1278802	−5.98 ± 56434
B	0.9770 ± 963950	0.99 ± 494776	−6.01 ± 211757
C	10.28 ± 8530181	0.99 ± 73080	−6.00 ± 328416
D	99.43 ± 109608398	0.99 ± 10893	−6.00 ± 473965
E	1074 ± 558660685	0.98 ± 476	−6.00 ± 225560
**Global fit with shared E**_**MAX**_			
A	0.1033 ± 0.0297	0.99 ± 0.01	−5.98 ± 0.58
B	0.9872 ± 0.0898	0.99 ± 0.01	−6.01 ± 0.12
C	11.12 ± 3.08	0.99 ± 0.01	−5.97 ± 0.14
D	224 ± 683	0.99 ± 0.01	−5.66 ± 1.32
E	971 ± 3064	0.99 ± 0.01	−6.03 ± 1.37
**Global fit with shared E**_**MAX**_ **and K**_**A**_			
A	0.1026 ± 0.0237	0.99 ± 0.01	−5.99 ± 0.09
B	0.9910 ± 0.0847	0.99 ± 0.01	−5.99 ± 0.09
C	10.64 ± 2.01	0.99 ± 0.01	−5.99 ± 0.09
D	102.5 ± 24.2	0.99 ± 0.01	−5.99 ± 0.09
E	1084 ± 259	0.99 ± 0.01	−5.99 ± 0.09

Simulation parameters log K_A_ = −6, E_MAX_ = 1; curve A, τ = 0.1; curve B, τ = 1; curve C, τ = 10; curve D, τ = 100; curve B, τ = 1000. Values are parameter estimates ± SD.

It is a requisite to check the distribution of estimated parameters before proceeding with statistical analysis. In pharmacological calculations, many parameters need to be converted to logarithm to remove skewness. To analyse the distribution of parameter τ, 1000 sets of concentration-response curves were simulated. Equation (1) was fitted to these datasets in two forms, one with parameter τ and another with parameter log(τ) (Supplementary information, Fig. SI2). In contrast to previous findings^7^, analysis of τ and log(τ) distribution has shown that whether to use τ or log(τ) in Eq. (1) is irrelevant.

As simulated data are for the same system they share parameter E_MAX_. Global fit of all 5 datasets with shared E_MAX_ reduced fit error (Table 1). The standard deviation of E_MAX_ fell down to 1%. However, SD of parameter τ was still several hundreds of % for datasets D and E. As simulated data are modelled for one agonist, K_A_ is a shared parameter of all datasets too. Global fit of all 5 datasets with shared E_MAX_ and K_A_ parameters further reduced fit error, SD of E_MAX_ remained at 1%, SD of K_A_ was 0.09 log unit and SD of operational efficacy τ was lowest for dataset B (8.5%) and gradually increased with changes in τ value in both directions up to 24%. This confirms previous findings showing the necessity to fit the OM simultaneously to at least two concentration-response curves with shared parameters^6^.

In practice, experimenter may determine agonist affinity for the receptor in a different kind of experiments, e.g. radioligand binding. The impact of fixing K_A_ to simulated value is summarized in Table 2. Fixing K_A_ to a correct value tremendously reduced fit error. For datasets C, D and E with the highest operational efficacy, SD of E_MAX_ was 1% and SD of parameter τ was about 6%. On the other hand, for dataset A with the lowest efficacy, SD of E_MAX_ was 54% and SD of parameter τ was 68%. According to the OM, EC_50_ is related to K_A_ according to the following equation.

2 $${{\rm{EC}}}_{{\rm{50}}}=\frac{{K}_{A}}{{\rm{\tau }}+1}$$

**Table 2:** Fitting OM with fixed K_A_ to simulated data.

Dataset	τ	E_MAX_	log K_A_
**Individual fits**			
A	0.0844 ± 0.0571	1.00 ± 0.54	−6
B	1.025 ± 0.096	0.97 ± 0.05	−6
C	10.32 ± 0.71	0.99 ± 0.01	−6
D	99.43 ± 6.80	0.99 ± 0.01	−6
E	1077 ± 52	0.98 ± 0.01	−6
**Global fit with shared E**_**MAX**_			
A	0.1024 ± 0.0223	0.99 ± 0.01	−6
B	0.9869 ± 0.0641	0.99 ± 0.01	−6
C	10.50 ± 0.93	0.99 ± 0.01	−6
D	100.8 ± 9.9	0.99 ± 0.01	−6
E	1065 ± 105	0.99 ± 0.01	−6

Simulation parameters log K_A_ = −6, E_MAX_ = 1; curve A, τ = 0.1; curve B, τ = 1; curve C, τ = 10; curve D, τ = 100; curve B, τ = 1000. Values are parameter estimates ± SD.

According to Eq. (2), higher operational efficacy τ means lower EC_50_, i.e., a greater left-ward shift of EC_50_ from K_A_. Because the accuracy of determination of EC_50_ is constant (on a logarithmic scale), the inaccuracy in EC_50_ determination represents proportionally greater error in low efficacy systems, where the difference between EC_50_ and K_A_ is small. Operational efficacy τ is bound to E_MAX_. Thus, the proportionally large error in estimation of operational efficacy τ is translated to a large error in estimation of E_MAX_. Fitting of the OM to all 5 datasets with fixed K_A_ and shared E_MAX_ led to an increase in the τ SD for datasets D and E. Although the increase in SDs after reducing the number of fitted parameters may seem counter-intuitive at first glance, it could be explained by the approach of E’_MAX_ to system E_MAX_. Moreover, the increase in τ SD for datasets D and E is compensated by the reduction of τ SD for datasets A and B.

Although fixing K_A_ in the model system results in estimates of E_MAX_ and τ that are close to the model values, the fitting procedure is still not robust. This is evidenced by the high SD values of E_MAX_ and τ estimates for dataset A (Table 2). When fitting Eq. (1) with fixed K_A_ and shared E_MAX_, SD of E_MAX_ falls to 1% but SD of parameter τ remains relatively high. Furthermore, to obtain correct K_A_, the experimenter must replicate the same conditions of functional response assay in the ligand binding assay. This may be technically difficult when functional assay is carried out at complicated system that is incompatible with radioligand binding assay. Therefore, we searched for an alternative way to obtain the value of all 3 parameters of the OM without a need to fit equation(s) that contain inter-bound parameters. The apparent maximal response E’_MAX_ observed as the upper asymptote of the functional response curve is given by the following equation.

3 $${{\rm{E}}^{\prime} }_{{\rm{MAX}}}=\frac{{E}_{{\rm{MAX}}}\ast \tau }{{\rm{\tau }}+1}$$

By rearrangement of Eq. (2), τ can be expressed as follows.

4 $$\tau =\frac{{K}_{A}-{{\rm{EC}}}_{{\rm{50}}}}{{{\rm{EC}}}_{50}}$$

Substitution of τ in Eq. (3) results in:

5 $${{\rm{E}}^{\prime} }_{{\rm{MAX}}}=\frac{\frac{{E}_{{\rm{MAX}}}\ast {K}_{A}-{{\rm{EC}}}_{{\rm{50}}}}{{{\rm{EC}}}_{{\rm{50}}}}}{\frac{{K}_{A}-{{\rm{EC}}}_{50}}{{{\rm{EC}}}_{{\rm{50}}}}+1}$$

After rearrangement and simplification of Eq. (5) we get:

6 $${{\rm{E}}^{\prime} }_{{\rm{MAX}}}={E}_{{\rm{MAX}}}-\frac{{E}_{{\rm{MAX}}}\ast {{\rm{EC}}}_{{\rm{50}}}}{{K}_{A}}$$

Apparent maximal response E’_MAX_ and half-efficient concentration EC_50_ can be reliably obtained by fitting logistic function (Eq. (10), see Methods) to individual concentration-response curves. After plotting E’_MAX_ versus the corresponding EC_50_, fitting Eq. (6) yields a maximal response of the system (E_MAX_) and the equilibrium dissociation constant of the agonist-receptor complex (K_A_).

Simulated data (Fig. 1, left) were fitted with Eq. (10) and resulting E’_MAX_ values were plotted against corresponding EC_50_ values (Fig. 1, right). Fitting Eq. (6) to the data yielded system E_MAX_ = 0.98 ± 0.01 and logarithm of the equilibrium dissociation constant of the agonist-receptor complex K_A_ = −5.99 ± 0.01 (parameter estimate ± SD). Subsequently, the OM of functional response Eq. (1) with E_MAX_ fixed to 0.98 and log K_A_ fixed to −5.99 was fitted to simulated data (Fig. 1, left, yellow curves). The standard deviation of the operational efficacy parameter τ ranged from less than 1‰ for dataset A to 6% for dataset D, that is a substantial improvement over fitting of the OM Eq. (1) with shared E_MAX_ and fixed K_A_ (Table 3 vs. Table 2).

**Table 3:** Fitting OM with fixed K_A_ and E_MAX_ to simulated data.

Dataset	τ	E_MAX_	log K_A_
**Individual fits**			
A	0.1036 ± 0.0009	0.98	−5.99
B	1.008 ± 0.013	0.98	−5.99
C	11.04 ± 0.48	0.98	−5.99
D	106.1 ± 6.7	0.98	−5.99
E	1119 ± 50	0.98	−5.99

Simulation parameters log K_A_ = −6, E_MAX_ = 1; curve A, τ = 0.1; curve B, τ = 1; curve C, τ = 10; curve D, τ = 100; curve B, τ = 1000. K_A_ and E_MAX_ were fixed to values obtained from E’_MAX_ versus EC_50_ plot (Fig. 1, right). Values are parameter estimates ± SD.

### Binding experiments

Five cell lines with various expression levels were chosen from newly established cell lines expressing fusion proteins of muscarinic receptor and the G_15_ G-protein. The expression level was determined in saturation binding experiments (Table 4). The expression level ranged from to 0.87 to 11.4 pmol of binding sites per mg of protein for the M_2__G_15_ fusion protein and from to 0.53 to 14.6 pmol of binding sites per mg of protein for the M_4__G_15_ fusion protein, respectively (Table 4). Expression level was stable among passages. Expression level had no effect on the equilibrium dissociation constant K_D_ of N-[^3^H]methylscopolamine ([^3^H]NMS). Fusion of G-protein to receptor resulted in a slightly lower K_D_ (higher affinity) of [^3^H]NMS than the wild-type receptor^8^. Inhibition constants K_I_ of carbachol, oxotremorine and pilocarpine, respectively, were determined in competition experiments with 1 nM [^3^H]NMS. All 3 agonists displayed binding to a single low-affinity site. Expression level had no effect on the equilibrium dissociation constant K_I_ of any of the tested agonists (Table 4).

**Table 4:** Binding parameters of fusion proteins of muscarinic receptor and G_15_ G-protein in CHO cell lines.

Cell line	[^3^H]NMS		carbachol	oxotremorine	pilocarpine
	pK_D_	B_MAX_	pK_I_	pK_I_	pK_I_
M_2__G_15_ #1	9.56 ± 0.02	11.4 ± 0.5	4.63 ± 0.02	5.72 ± 0.03	4.49 ± 0.03
M_2__G_15_ #2	9.56 ± 0.02	5.40 ± 0.09	4.61 ± 0.02	5.76 ± 0.03	4.52 ± 0.02
M_2__G_15_ #3	9.55 ± 0.02	2.52 ± 0.05	4.64 ± 0.02	5.78 ± 0.03	4.51 ± 0.02
M_2__G_15_ #4	9.57 ± 0.02	1.82 ± 0.03	4.63 ± 0.02	5.75 ± 0.02	4.54 ± 0.03
M_2__G_15_ #5	9.55 ± 0.02	0.87 ± 0.01	4.64 ± 0.02	5.73 ± 0.03	4.50 ± 0.02
M_4__G_15_ #1	9.72 ± 0.02	14.6 ± 0.7	4.63 ± 0.02	5.86 ± 0.03	4.55 ± 0.03
M_4__G_15_ #2	9.73 ± 0.02	6.02 ± 0.08	4.65 ± 0.02	5.84 ± 0.03	4.54 ± 0.02
M_4__G_15_ #3	9.72 ± 0.02	2.11 ± 0.06	4.62 ± 0.02	5.83 ± 0.03	4.51 ± 0.03
M_4__G_15_ #4	9.71 ± 0.02	1.33 ± 0.04	4.63 ± 0.02	5.80 ± 0.03	4.53 ± 0.02
M_4__G_15_ #5	9.75 ± 0.03	0.53 ± 0.02	4.62 ± 0.02	5.82 ± 0.03	4.52 ± 0.02

Equilibrium dissociation constant (K_D_) and maximum binding capacity (B_MAX_) were obtained by fitting Eq. (7) to the data from [^3^H]NMS saturation experiments. B_MAX_ is expressed as pmol binding sites per mg of protein. Inhibition constants (K_I_) were calculated according Eq. (9) from IC_50_ values that were obtained by fitting Eq. (8) to the data from competition experiments of agonist and [^3^H]NMS. Data are means ± SD from 3 independent experiments performed in quadruplicates.

### Functional response to agonists

**Figure 2:**
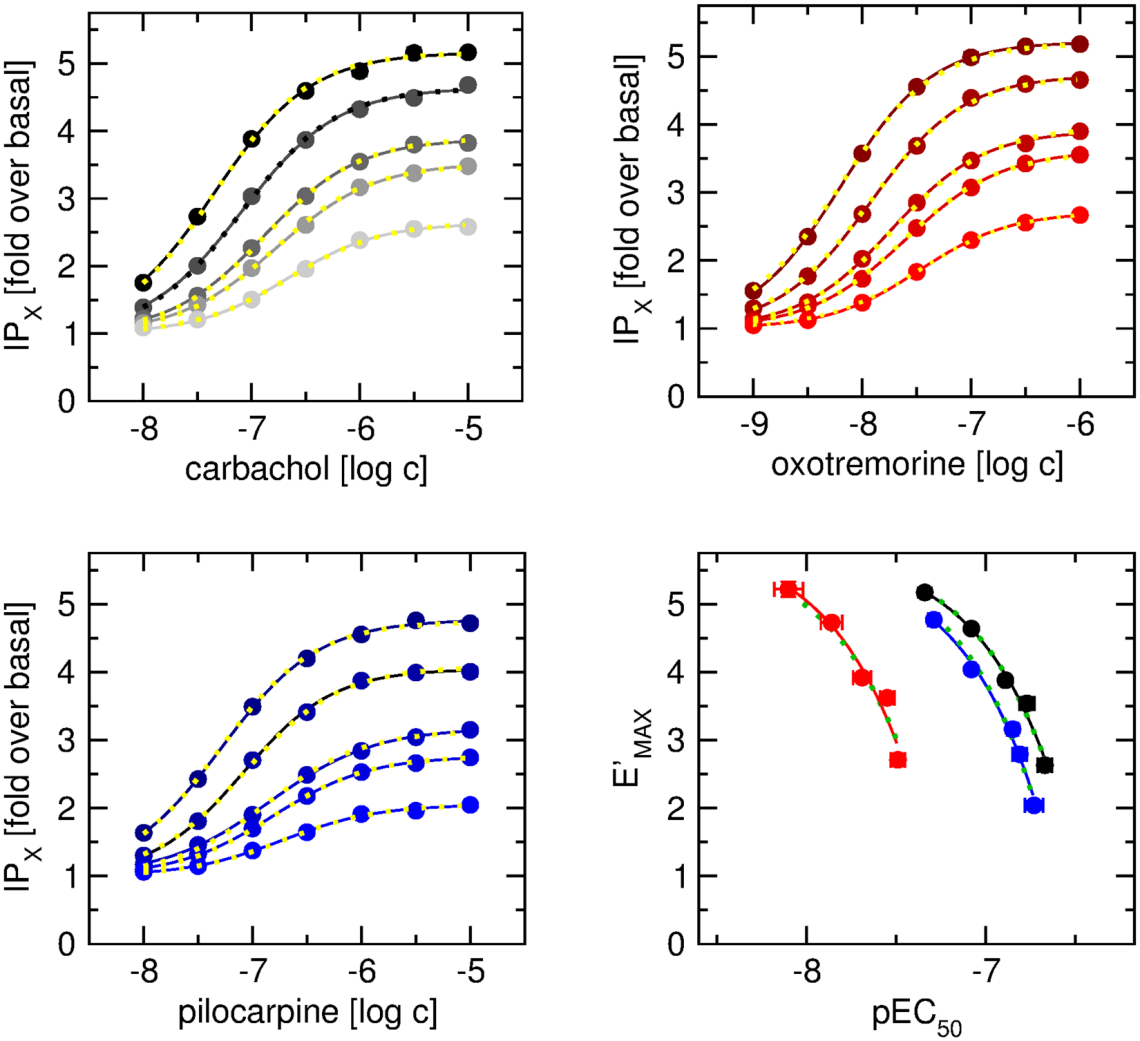
Fitting of the OM to concentration response curves in cells expressing M_2__G_15_ fusion protein. Level of inositol phosphates (IP_X_) was taken as the functional response measure and is expressed as folds over basal level (16 ± 1% of incorporated radioactivity). Eq. (10) was fitted to data of functional response (solid lines) to carbachol (upper left), oxotremorine (upper right) and pilocarpine (lower left) at cells expressing M_2__G_15_ fusion protein at various levels. After subtraction of basal value Eq. (6) was fitted to E’_MAX_ versus EC_50_ plot (lower right) of functional response to carbachol (black), oxotremorine (red) and pilocarpine (blue) to obtain E_MAX_ and K_A_ values, solid lines – individual fits, green dotted lines – shared E_MAX_ fit. Eq. (1) with fixed E_MAX_ and K_A_ parameters was fitted to data of functional response (dotted yellow lines). Data are means ± SD from 3 independent experiments performed in quadruplicates.

**Figure 3:**
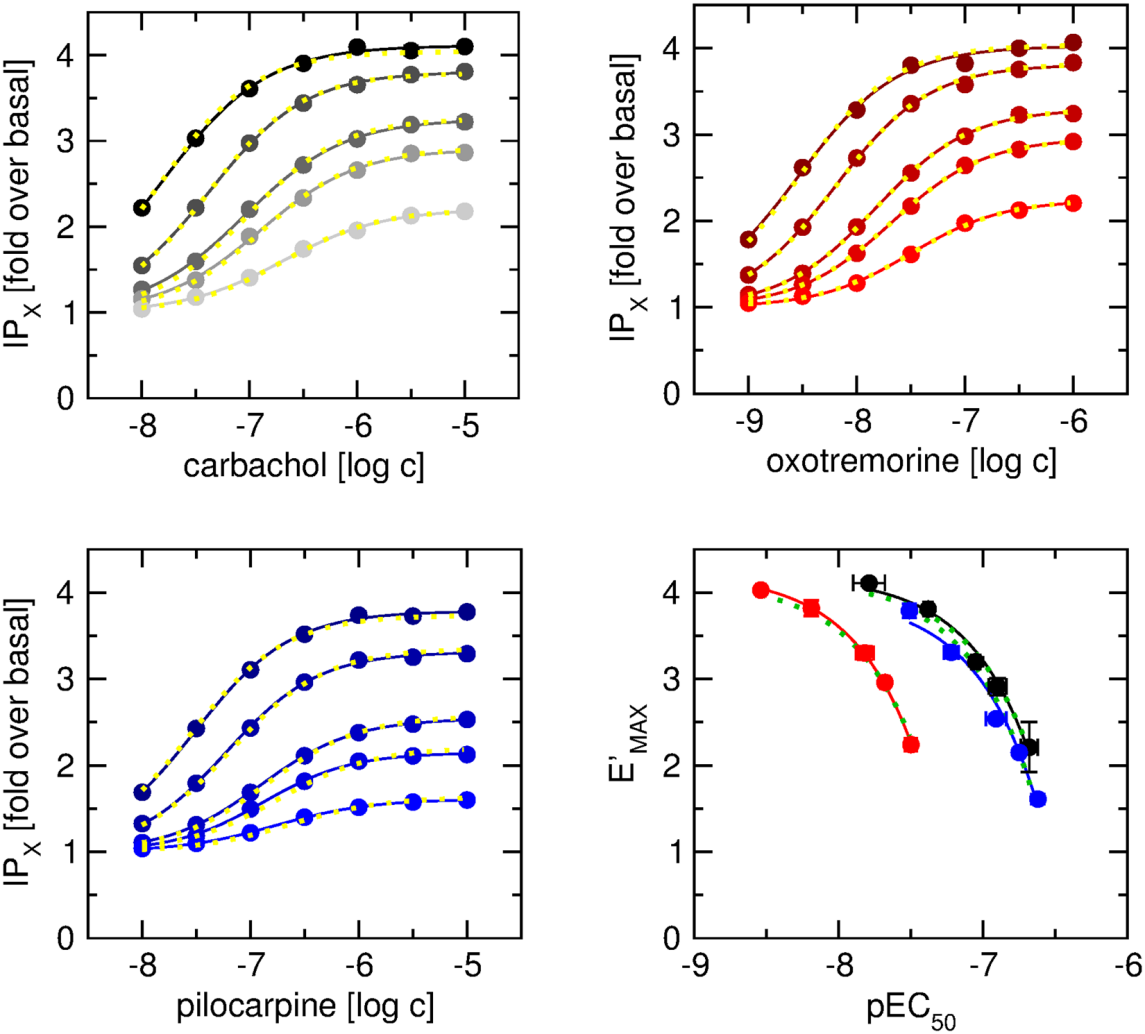
Fitting of the OM to concentration response curves in cells expressing M_4__G_15_ fusion protein. Level of inositol phosphates (IP_X_) was taken as functional response measure and is expressed as folds over basal level (21 ± 1% of incorporated radioactivity). Eq. (10) was fitted to data of functional response (solid lines) to carbachol (upper left), oxotremorine (upper right) and pilocarpine (lower left) at cells expressing M_2__G_15_ fusion protein at various levels. After subtraction of basal value Eq. (6) was fitted to E’_MAX_ versus EC_50_ plot (lower right) of functional response to carbachol (black), oxotremorine (red) and pilocarpine (blue) to obtain E_MAX_ and K_A_ values, solid lines – individual fits, green dotted lines – shared E_MAX_ fit. Eq. (1) with fixed E_MAX_ and K_A_ parameters was fitted to data of functional response (dotted yellow lines). Data are means ± SD from 3 independent experiments performed in quadruplicates.

M_2_ and M_4_ receptors were coupled to the phospholipase C pathway by G_15_ G-protein^9^. The level of inositol phosphates (IP_X_) was taken as a measure of functional response. Response to agonists was plotted as folds over basal (Figs 2 and 3). Analysis of the theoretical model of the OM fitting of Eq. (1) to individual response curves resulted in expected large errors in estimation of fitted parameters. Unfortunately, values of inhibition constants (K_I_) obtained from binding experiments could not be used as K_A_ values in the fitting of Eq. (1) to the functional response data (Supplementary Information, Figs SI3–SI8). In fitting individual response curves, fixing K_A_ to K_I_ value resulted in good fit with high values of τ and variation in E_MAX_ estimates (Supplementary Information, Figs SI3–SI8, full lines). Fitting response curves with shared E_MAX_ resulted in poor fits (Supplementary Information, Figs SI3–SI8, dotted yellow lines). According to Eq. (4), large (100 to 1000-fold) differences between K_I_ and half-efficient concentrations (EC_50_) correspond to large (l00 to 1000) values of τ. According to Eq. (3), for very large values of τ the apparent maximal response E’_MAX_ should be very close to the maximal response of the system E_MAX_. Therefore, apparent E’_MAX_ should theoretically be the same for all receptor expression levels. Obviously, this is not the case here (Figs 2 and 3).

Therefore, a new fitting procedure was employed in which maximal response of the system (E_MAX_) and agonist equilibrium dissociation constant (K_A_) were determined first and then E_MAX_ and K_A_ values were used in fitting of the OM to the functional response data. Specifically, Eq. (10) was fitted to the data and apparent maximal response (E’_MAX_), half-efficient concentration (EC_50_) and slope factor (nH) were determined. All concentration-response curves displayed nH equal to unity. Expression level affected both E’_MAX_ and EC_50_. Increase in expression level resulted in an increase in E’_MAX_ and a decrease in EC_50_. The magnitude of effects of expression level on functional response at M_2__G_15_ was similar to that at M_4__G_15_. Calculated E’_MAX_ values were plotted against corresponding EC_50_ values (Figs 2 and 3, right bottom plots) and Eq. (6) was fitted to the data. Fitting of Eq. (6) to experimental data resulted in the same E_MAX_ for all 3 agonists confirming that the cell clones had the same E_MAX_. Resulting E_MAX_ and K_A_ values were used in the subsequent fitting of Eq. (1) to the functional response data (after subtraction of basal level). For a given agonist global fit of Eq. (1) was performed in all 5 cell lines (Figs 2 and 3, yellow lines). Calculated values of the operational efficacy factor (τ) are summarized in Tables 5 and 6.

**Table 5:** Parameters of functional response of fusion protein of M_2_ muscarinic receptor and G_15_ G-protein in CHO cell lines.

	carbachol	oxotremorine	pilocarpine
E_MAX_	5.81 ± 0.12	5.77 ± 0.14	5.68 ± 0.10
pK_A_	6.47 ± 0.04	7.28 ± 0.03	6.60 ± 0.02
**τ**			
M_2__G_15_ #1	6.75 ± 0.20	6.93 ± 0.21	1.73 ± 0.03
M_2__G_15_ #2	3.22 ± 0.18	3.43 ± 0.09	1.44 ± 0.01
M_2__G_15_ #3	1.50 ± 0.04	2.59 ± 0.04	0.781 ± 0.011
M_2__G_15_ #4	1.08 ± 0.02	1.47 ± 0.02	0.555 ± 0.005
M_2__G_15_ #5	0.521 ± 0.008	0.578 ± 0.008	0.199 ± 0.006

Maximal response of the system (E_MAX_) and equilibrium dissociation constant of the agonist-receptor complex (K_A_) were calculated by fitting Eq. (6) to E’_MAX_ versus EC_50_ plot in Fig. 2. The operational factor of efficacy (τ) was obtained by fitting Eq. (1) with fixed parameters E_MAX_ and K_A_ to concentration response curves in Fig. 2. Data are means ± SD from 3 independent experiments performed in quadruplicates.

**Table 6:** Parameters of functional response of fusion protein of M_4_ muscarinic receptor and G_15_ G-protein in CHO cell lines.

	carbachol	oxotremorine	pilocarpine
E_MAX_	4.19 ± 0.08	4.22 ± 0.02	4.03 ± 0.14
pK_A_	6.48 ± 0.04	7.28 ± 0.02	6.53 ± 0.01
**τ**			
M_4__G_15_ #1	15.7 ± 0.4	17.0 ± 0.5	9.41 ± 0.50
M_4__G_15_ #2	7.02 ± 0.38	7.54 ± 0.34	3.48 ± 0.15
M_4__G_15_ #3	2.46 ± 0.25	2.49 ± 0.08	1.08 ± 0.02
M_4__G_15_ #4	1.55 ± 0.12	1.64 ± 0.04	0.66 ± 0.01
M_4__G_15_ #5	0.720 ± 0.097	0.835 ± 0.015	0.265 ± 0.013

Maximal response of the system (E_MAX_) and equilibrium dissociation constant of the agonist-receptor complex (K_A_) were calculated by fitting Eq. (6) to E’_MAX_ versus EC_50_ plot in Fig. 3. The operational factor of efficacy (τ) was obtained by fitting Eq. (1) with fixed parameters E_MAX_ and K_A_ to concentration response curves in Fig. 3. Data are means ± SD from 3 independent experiments performed in quadruplicates.

Basal activity of M_2_ and M_4_ fusion proteins was 16 ± 1 and 21 ± 1% of incorporated radioactivity (mean ± SD, n = 3), respectively (Figs 2 and 3). E_MAX_ estimated according to Eq. (6) ranged from 91 to 93% of incorporated radioactivity at M_2_ and from 85 to 89% of incorporated radioactivity at M_4_ fusion protein.

**Figure 4:**
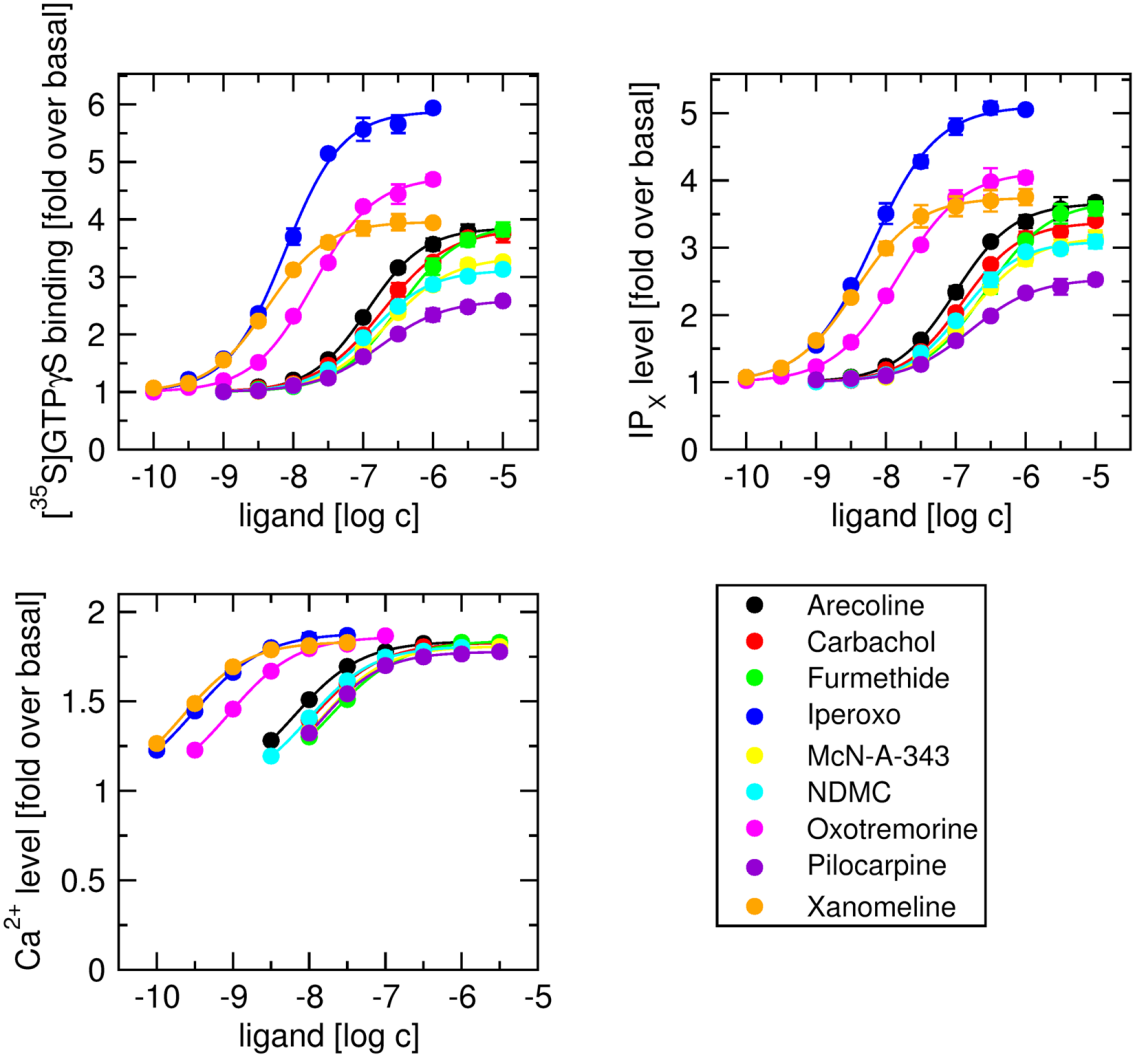
Functional response of cells expressing M_2__G_15_ fusion protein to muscarinic agonists. Level of [^35^S]GTPγS binding (upper left), inositol phosphates (IP_X_) (upper right) and intracellular calcium (lower left) was taken as functional response to muscarinic agonists (indicated in legend) of cells expressing M_2__G_15_ fusion protein and is expressed as folds over basal level. Eq. (10) was fitted to individual data sets. After subtraction of basal value Eq. (6) was fitted to E’_MAX_ versus EC_50_ data from all 3 experiments for all agonists to obtain E_MAX_ of the assay. Eq. (1) with fixed E_MAX_ was fitted to individual functional responses. Results are summarized in Table 7. Data are means ± SD from 3 independent experiments.

**Figure 5:**
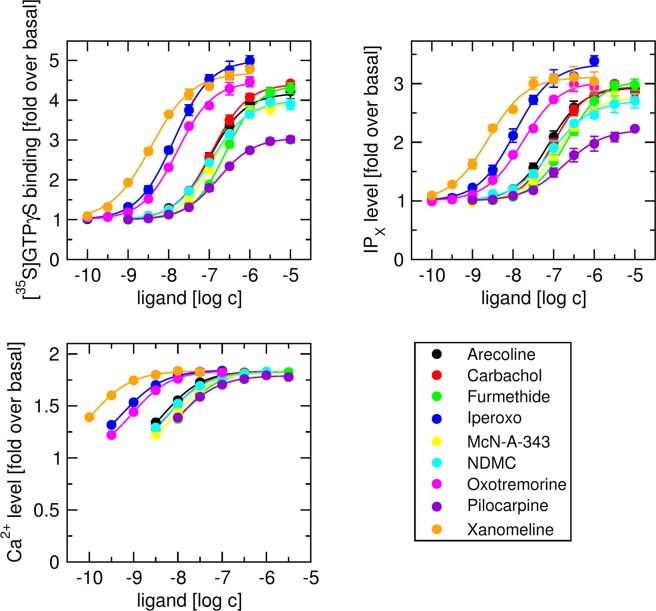
Functional response of cells expressing M_4__G_15_ fusion protein to muscarinic agonists. Level of [^35^S]GTPγS binding (upper left), inositol phosphates (IP_X_) (upper right) and intracellular calcium (lower left) was taken as functional response to muscarinic agonists (indicated in legend) of cells expressing M_4__G_15_ fusion protein and is expressed as folds over basal level. Eq. (10) was fitted to individual data sets. After subtraction of basal value Eq. (6) was fitted to E’_MAX_ versus EC_50_ data from all 3 experiments for all agonists to obtain E_MAX_ of the assay. Eq. (1) with fixed E_MAX_ was fitted to individual functional responses. Results are summarized in Table 8. Data are means ± SD from 3 independent experiments.

Subsequently, functional responses to 9 muscarinic agonists in cell clones with medium expression of fusion protein (M_2__G_15_#3 and M_4__G_15_#3) was measured in three ways: As stimulation of [^35^S]GTPγS binding, as accumulation of IP_X_, and as changes in the level of intracellular calcium (Figs 4 and 5; Tables 7 and 8). Muscarinic agonists included classical full and partial agonists, the superagonist iperoxo. the bitopic agonist McN-A-343 and the atypical agonists N-desmethylclozapine and xanomeline. First, Eq. (10) was fitted to individual data sets. After subtraction of basal value, Eq. (6) was fitted to E’_MAX_ versus EC_50_ data from all 3 experiments for all agonists to obtain E_MAX_ of the assay. Then Eq. (1) with fixed E_MAX_ was fitted to individual functional responses. In IP_X_ assay, τ values of the agonists carbachol, oxotremorine and pilocarpine were the same as in simultaneous fitting of OM to all five clones where both K_A_ and E_MAX_ were fixed (Figs 2 and 3; Tables 5 and 6). However, estimates of parameter τ were accompanied by greater SD. In [^35^S]GTPγS binding and IP_X_ assays the functional response even to the superagonist iperoxo was less than 90% at M_2__G_15_ and less than 80% at M_4__G_15_. In these assays relative SD of τ was about 10%. In intracellular Ca^2+^ mobilisation assay the functional response to iperoxo reached 99% of E_MAX_. In this assay relative SD of τ ranged from 15 to 34%, being much lower than that in case of direct fitting of OM with shared E_MAX_. In intracellular Ca^2+^ mobilisation assay, agonist ranking by parameter τ was the same as in [^35^S]GTPγS and IP_X_ assays. Also, the estimates of K_A_ were the same in all assays.

**Table 7:** Parameters of functional response of M_2__G_15_ fusion protein to muscarinic agonists.

	pEC_50_	E’_MAX_	pK_A_	τ
***[***^***35***^***S]GTPγS binding***, ***E***_***MAX***_ = ***7***.***33***				
Arecoline	6.94 ± 0.03	3.8 ± 0.2	6.6 ± 0.1	1.09 ± 0.08
Carbachol	6.77 ± 0.03	3.7 ± 0.2	6.5 ± 0.1	1.06 ± 0.08
Furmethide	6.48 ± 0.03	3.9 ± 0.2	6.1 ± 0.1	1.17 ± 0.09
Iperoxo	8.09 ± 0.02	5.9 ± 0.3	7.4 ± 0.1	4.3 ± 0.4
McN-A-343	6.70 ± 0.03	3.3 ± 0.2	6.4 ± 0.1	0.80 ± 0.06
NDMC	6.84 ± 0.03	3.2 ± 0.2	6.6 ± 0.1	0.79 ± 0.06
Oxotremorine	7.73 ± 0.02	4.7 ± 0.3	7.3 ± 0.1	1.75 ± 0.14
Pilocarpine	6.80 ± 0.03	2.6 ± 0.1	6.6 ± 0.1	0.54 ± 0.04
Xanomeline	8.34 ± 0.02	4.1 ± 0.2	8.0 ± 0.1	1.24 ± 0.10
***IP***_***X***_ ***level***, ***E***_***MAX***_ = ***5***.***75***				
Arecoline	7.02 ± 0.02	3.6 ± 0.2	6.6 ± 0.1	1.67 ± 0.15
Carbachol	6.87 ± 0.03	3.5 ± 0.2	6.5 ± 0.1	1.50 ± 0.13
Furmethide	6.57 ± 0.03	3.7 ± 0.2	6.1 ± 0.1	1.78 ± 0.16
Iperoxo	8.23 ± 0.02	5.0 ± 0.3	7.4 ± 0.1	6.7 ± 0.7
McN-A-343	6.77 ± 0.03	3.2 ± 0.2	6.4 ± 0.1	1.21 ± 0.09
NDMC	6.88 ± 0.03	3.1 ± 0.2	6.5 ± 0.1	1.19 ± 0.08
Oxotremorine	7.84 ± 0.02	4.2 ± 0.2	7.3 ± 0.1	2.59 ± 0.21
Pilocarpine	6.85 ± 0.02	2.5 ± 0.1	6.6 ± 0.1	0.78 ± 0.06
Xanomeline	8.46 ± 0.02	3.7 ± 0.2	8.0 ± 0.1	1.83 ± 0.15
***Intracellular Ca***^***2+***^ ***level***, ***E***_***MAX***_ = ***1***.***879***				
Arecoline	8.19 ± 0.02	1.828 ± 0.003	6.6 ± 0.1	35 ± 5
Carbachol	7.96 ± 0.03	1.829 ± 0.003	6.4 ± 0.1	33 ± 5
Furmethide	7.73 ± 0.03	1.830 ± 0.003	6.1 ± 0.1	38 ± 6
Iperoxo	9.53 ± 0.02	1.839 ± 0.003	7.3 ± 0.1	156 ± 51
McN-A-343	7.85 ± 0.03	1.820 ± 0.003	6.4 ± 0.1	27 ± 5
NDMC	8.01 ± 0.03	1.816 ± 0.003	6.6 ± 0.1	26 ± 4
Oxotremorine	9.07 ± 0.02	1.831 ± 0.003	7.3 ± 0.1	64 ± 14
Pilocarpine	7.85 ± 0.02	1.791 ± 0.003	6.6 ± 0.1	18 ± 3
Xanomeline	9.65 ± 0.02	1.834 ± 0.003	8.0 ± 0.1	44 ± 8

Eq. (10) was fitted to individual data sets. Eq. (6) was fitted to E’_MAX_ versus EC_50_ data from all 3 experiments for all agonists to obtain E_MAX_ of the assay. Equation (1) with fixed E_MAX_ was fitted to individual functional responses. Data are means ± SD from 3 independent experiments.

**Table 8:** Parameters of functional response of M_4__G_15_ fusion protein to muscarinic agonists.

	pEC_50_	E’_MAX_	pK_A_	τ
***[***^***35***^***S]GTPγS binding***, ***E***_***MAX***_ = ***6***.***85***				
Arecoline	6.98 ± 0.03	4.2 ± 0.2	6.6 ± 0.1	1.60 ± 0.12
Carbachol	6.94 ± 0.03	4.3 ± 0.2	6.5 ± 0.1	1.74 ± 0.13
Furmethide	6.58 ± 0.03	4.4 ± 0.2	6.1 ± 0.1	1.77 ± 0.13
Iperoxo	7.88 ± 0.02	5.0 ± 0.3	7.3 ± 0.1	2.70 ± 0.22
McN-A-343	6.86 ± 0.03	4.0 ± 0.2	6.5 ± 0.1	1.43 ± 0.12
NDMC	7.01 ± 0.03	3.9 ± 0.2	6.6 ± 0.1	1.32 ± 0.11
Oxotremorine	7.77 ± 0.02	4.4 ± 0.2	7.3 ± 0.1	1.84 ± 0.13
Pilocarpine	6.78 ± 0.03	3.0 ± 0.2	6.5 ± 0.1	0.80 ± 0.06
Xanomeline	8.50 ± 0.02	4.6 ± 0.2	8.0 ± 0.1	2.06 ± 0.16
***IP***_***X***_ ***level***, ***E***_***MAX***_ = ***4***.***18***				
Arecoline	7.10 ± 0.02	2.9 ± 0.1	6.6 ± 0.1	2.37 ± 0.22
Carbachol	7.02 ± 0.03	3.0 ± 0.2	6.5 ± 0.1	2.45 ± 0.23
Furmethide	6.67 ± 0.03	3.0 ± 0.2	6.1 ± 0.1	2.57 ± 0.25
Iperoxo	7.97 ± 0.02	3.3 ± 0.2	7.3 ± 0.1	3.9 ± 0.4
McN-A-343	6.92 ± 0.03	2.8 ± 0.1	6.5 ± 0.1	1.96 ± 0.18
NDMC	7.10 ± 0.03	2.7 ± 0.1	6.7 ± 0.1	1.79 ± 0.16
Oxotremorine	7.82 ± 0.02	3.0 ± 0.2	7.3 ± 0.1	2.48 ± 0.24
Pilocarpine	6.85 ± 0.02	2.2 ± 0.1	6.5 ± 0.1	1.08 ± 0.09
Xanomeline	8.62 ± 0.02	3.1 ± 0.2	8.0 ± 0.1	2.77 ± 0.28
***Intracellular Ca***^***2+***^ ***level***, ***E***_***MAX***_ = ***1***.***861***				
Arecoline	8.32 ± 0.02	1.828 ± 0.003	6.6 ± 0.1	57 ± 10
Carbachol	8.22 ± 0.03	1.829 ± 0.003	6.4 ± 0.1	59 ± 10
Furmethide	7.93 ± 0.03	1.830 ± 0.003	6.1 ± 0.1	61 ± 11
Iperoxo	9.27 ± 0.02	1.839 ± 0.003	7.3 ± 0.1	87 ± 29
McN-A-343	8.08 ± 0.03	1.820 ± 0.003	6.4 ± 0.1	45 ± 5
NDMC	8.24 ± 0.03	1.816 ± 0.003	6.6 ± 0.1	41 ± 6
Oxotremorine	9.05 ± 0.02	1.831 ± 0.003	7.2 ± 0.1	63 ± 11
Pilocarpine	8.00 ± 0.02	1.791 ± 0.003	6.6 ± 0.1	26 ± 4
Xanomeline	9.93 ± 0.02	1.834 ± 0.003	8.1 ± 0.1	71 ± 14

Equation (10) was fitted to individual data sets. Equation (6) was fitted to E’_MAX_ versus EC_50_ data from all 3 experiments for all agonists to obtain E_MAX_ of the assay. Equation (1) with fixed E_MAX_ was fitted to individual functional responses. Data are means ± SD from 3 independent experiments.

## Discussion

Proper determination of agonist efficacy is a cornerstone in the assessment of possible agonist selectivity and signalling bias. Agonist efficacy at a given receptor or receptor subtype is affected by the system in which it is determined. Black and Leff^1^ presented a model, termed the operational model of agonism (OM) of receptor-effector systems. In its original principle, the OM is applicable to any receptor effector system. Over time the OM became a golden standard in pharmacological analysis^3,4^. Extended and modified versions of this model are widely applied, not only to agonist action, but also to allosteric activation and allosteric modulation^10–14^ or to analyse agonist signalling bias at GPCRs^15–18^ as well as non-GPCRs^19^.

In the present work we explored potential pitfalls of application of the original OM to fit experimental data. We present a simple procedure for reliable fitting of the OM to experimental data. The procedure divides actual fitting of OM to two steps. Individual steps have lower number of degrees of freedom and thus result in more reliable parameter estimates. We demonstrate usefulness of this procedure in a system where the affinity of the agonist for the receptor in active state cannot be reliably determined in binding experiments and also in a system where the functional response to the agonist approaches the maximal response of the system.

Response to an agonist is affected by the system, mainly by the expression level of the receptor. Mass action law dictates that the relationship between occupancy and effect must be hyperbolic. Therefore, response to agonist according to the OM is described by Eq. (1), where E_MAX_ is the maximal response of the system, K_A_ is the agonist equilibrium dissociation constant at the receptor in an active conformation and τ is the operational efficacy^1^. All 3 parameters (E_MAX_, K_A_ and τ) of Eq. (1) are inter-bound and thus Eq. (1) should be fitted to at least two concentration-response curves with shared parameters^6^. The asymptote of the response curve is given by the product of τ and E_MAX_ and inflection point is given by the ratio of K_A_ to τ. Thus, K_A_ is also inter-bound to E_MAX_ via τ. Functions with inter-bound parameters generally have flat curvature of sum of error function that prevents finding its global minimum and estimation of fitted parameters is therefore associated with large error margins. This was confirmed by fitting of Eq. (1) to individual response curves of the theoretical model system (Fig. 1, Tables 1 and 2). Moreover, result of fit (parameter estimates) is influenced by initial values of fitted parameters (Supplementary Information, Fig. SI1). Further analysis of the theoretical model system showed that major improvement of fit is achieved by fixing K_A_ value while performing global fit with shared E_MAX_. Thus, precise determination of K_A_ turns to be corner stone in application of the OM. However, in contrast to previous findings^7^, analysis of parameter τ distribution was independent of whether τ or log(τ) is used (Supplementary information, Fig. SI2).

Experimental conditions (like temperature, ionic strength, solution composition, receptor environment) affect agonist K_A_. Thus, when K_A_ is determined in different kinds of experiment (e.g. radioligand binding assay), conditions of the assay have to strictly mimic those in functional assay. In this case, K_A_ should be determined at whole cells attached to the bottom of 96-well plate. In whole cells, agonists display low-affinity binding that is the result of negative cooperativity between binding of GDP and binding of an agonist to the receptor G-protein complex^20^. K_A_ is the ratio between the experimentally calculated low-affinity K_I_ and factor of binding cooperativity of GDP and agonist. As far as the factor of binding cooperativity is unknown, K_A_ cannot be calculated. In membranes, muscarinic agonists usually display both high- and low-affinity binding^21^ as free GDP is removed and bound GDP partly dissociates during membrane preparation. At the M_2__G_15_ and M_4__G_15_ fusion proteins, however, inhibition of [^3^H]NMS binding by tested agonists showed only low-affinity binding (Table 4). It is possible that at constructed fusion proteins interaction of G-protein with the receptor in an inactive conformation is very strong and that GDP is locked in its binding site at G-protein^22^. The locking precludes dissociation of GDP from the G-protein. An open question remains, namely whether K_I_ of agonist high-affinity binding observed in membranes can be taken as K_A_. Actually, K_A_ may be affected by many factors including binding cooperativity between agonist and GDP-less G-protein and by changes resulting from membrane preparation. Taking this into account, it may be actually safer to determine K_A_ from a series of functional experiments according to the procedure described below than to determine K_A_ in radioligand binding assays.

Indeed, inhibition constants K_I_ of low-affinity agonist binding were too high to be considered as K_A_. Considering that K_A_ equals K_I_ (Supplementary Information, Figs SI3–SI8) resulted in high values of τ as a result of high K_A_ to EC_50_ ratio (Eq, d). For high values of τ apparent maximal response E’_MAX_ approximates maximal response of the system E_MAX_ (Eq. (3)). Thus variation in E’_MAX_ caused variation in E_MAX_. However, E_MAX_ should be the same for all 5 cell clones. Global fit of Eq. (1) to functional response data in all 5 cell clones with shared E’_MAX_ did not converge (Supplementary Information, Figs SI3–SI8, yellow dotted lines).

Therefore, a novel procedure to obtain reliable values of K_A_ and E_MAX_ by meta-analysis of functional response data was developed. Analysis of the OM according to Eq. (1) shows that the apparent maximal response E’_MAX_ relates to the maximal response of the system E_MAX_ according to Eq. (3) and EC_50_ relates to K_A_ according to Eq. (4). E’_MAX_ and EC_50_ were obtained by fitting Eq. (10) to data of individual functional responses (Figs 2 and 3, left and right upper and left lower plots, full lines). Subsequently, E’_MAX_ values were taken as a function of corresponding EC_50_ values and parameters E_MAX_ and K_A_ were obtained by fitting Eq. (6) (Figs 2 and 3, right lower plot). Obtained E_MAX_ and K_A_ values (Tables 5 and 6) were used to globally fit Eq. (1) to functional response data (Figs 2 and 3, left and right upper and left lower plots, dotted yellow lines). With only one exception SD of τ estimates was well below 10% (Tables 5 and 6). That is a great improvement over direct fitting of Eq. (1) to the functional data (Supplementary Information, Figs SI3–SI8).

Another possibility how to reduce the number of degrees of freedom in the fitting of the OM to data is to fix E_MAX_. In case of accumulation of inositol phosphates, it may seem intuitive that 100% of incorporated radioactivity may be metabolized to inositol trisphosphate, and that is the actual maximal response of the system (E_MAX_). However, as shown by presented experimental data E_MAX_ is around 90% of incorporated radioactivity. This is given by the fact that converting substrate to product decreases substrate level and increases product level, resulting in feedback inhibition of the forward reaction. The level at which the reaction comes to equilibrium between substrate and product may be affected by various other factors (e.g. starting concentration of substrate, inhibition of subsequent metabolic steps, related metabolic processes). In case of [^35^S]GTPγS binding, the amount of GTPγS is greater than the amount of G-protein. Therefore, it may seem intuitive that 100% occupancy of G-protein represents the E_MAX_. However, E_MAX_ expressed as occupancy of G-protein in this assay is less than 20%. In case of intracellular calcium, E_MAX_ is given by the ratio of the rate of calcium release from intracellular stores to the rate of calcium clearance from cytoplasm. Thus, E_MAX_ cannot be established *a priori* and needs to be measured experimentally. Fitting Eq. (6) to E’_MAX_ versus EC_50_ data allows calculation of the E_MAX_ of the assay.

A drawback of the described procedure is that at least 3 systems (cell clones) with substantially different receptor expression level are needed to fit Eq. (6) to functional response meta-data for a given agonist. Having only 2 systems can be overcome by combining 3 or more agonists substantially differing in efficacy for a global fit of Eq. (6) to functional response meta-data (Figs 2 and 3, lower right plot, green dotted lines). Having only one system (Figs 4 and 5, Tables 7 and 8), the first step for ranking a batch of agonists in terms of efficacy is to fit Eq (10) to individual functional responses. After subtraction of basal value the relationship between E’_MAX_ and EC_50_ for all agonists was fitted to Eq. (6) to to obtain E_MAX_ of the assay. Finally, one fits Eq. (1) with fixed E_MAX_ to individual functional responses.

Regardless the number of systems employed, the procedure is limited to conditions with substantially different τ values ranging from 0.1 to 100. The procedure will not work when only agonists with τ greater than 100 are available as K_A_ could not be properly estimated using Eq. (6). Such situation should be resolved experimentally by reduction of receptor number using an irreversible antagonist that will bring τ values down to allow K_A_ determination. Similarly, if only very weak agonists with τ lower than 0.1 are available, then the procedure will not work either as system E_MAX_ could not be properly estimated using Eq. (6). In such case, a highly efficacious agonist needs to be added for comparison. Measurements of intracellular Ca^2+^ (Figs 4 and 5, Tables 7 and 8) show that our procedure can be used in a system where E’_MAX_ of agonists approaches E_MAX_ of the system; a case where direct fitting of OM would be problematic.

In principle, the procedure described above can be used everywhere OM is applicable including other G-protein coupled receptors or any receptor-effector system in general^1,2^. Moreover, the procedure can also be applied to all possible variants of the OM as far as relations between parameters of the OM variant and parameters of classical (e.g. logistic) function can be established. For example, introducing the slope factor (Hill coefficient) to Eq. (1) does not change the relation of EC_50_ to K_A_ and E’_MAX_ to E_MAX_ as slope factor cancels out itself in Eqs (2) and (3).

## Conclusions

Described two-step analysis of functional response represents the robust way of fitting operational model of agonism (OM) to experimental data. Although our procedure was developed using muscarinic acetylcholine receptors as a test system, it is in principle applicable to any receptor-effector system where OM is used. Also, the procedure can be applied to all possible variants of the OM as far as relations between parameters of the OM variant and parameters of classical (e.g. logistic) function can be established. We believe the procedure will have significant practical value in the proper ranking of agonists efficacies in various systems.

## Methods

### Receptor mutagenesis and generation of cell lines

New stable Chinese hamster ovary (CHO) cells lines expressing fusion proteins of M_2_ or M_4_ receptors and G_15_ G-protein were prepared. Plasmid pcDNA3.1 (Invitrogen) containing the coding sequence of the human variants of muscarinic receptors and that of the G_15_ G-protein were obtained from Missouri S&T cDNA Resource Center (Rolla, MO, USA). Mammalian vector with hygromycin as mammalian selection marker pCMV6-A-Hygro was purchased from Origene (Rockville, MD). First, the G_15_ G-protein coding sequence was subcloned into pCMV6-A-Hygro plasmid. Then AflII sites were generated at the 3′-end of coding sequence of M_2_ and M_4_ using Quick Change II Site-directed Mutagenesis Kit (Stratagene). The coding sequence of the receptor was subcloned into G_15_-pCMV-A-Hygro plasmid. The M_2_ coding sequence was ligated directly to the G_15_ coding sequence. In case of the M_4_ receptor, ARATR linker was inserted between the receptor and the G-protein (resulting sequences are in Supplementary Information). CHO-K1 cells were transfected with the desired plasmids using Lipofectamine 3000 (Invitrogen). Subconfluent cells were washed with phosphate-buffered saline and then Opti-MEM (Life Technologies) containing Lipofectamine at a final concentration of 1.5 μl/ml and plasmid DNA was applied at a final concentration of 0.5 μg/ml. After 48 hours cells were diluted 1000-times by subculturing and hygromycin-B (Toku-E) was added at final concentration of 200 ng/ml for selection of transfected clones. For the study 5 clones of each construct selected from the single selection were used up to passage 10. Expression of new constructs was checked by reverse transcription quantitative PCR. Mediator RNA was isolated from CHO cell lines using TriPure Isolation Reagent (Roche). Reverse transcription was done using M-MLV Reverse Transcriptase (Promega), Oligo (dT) anchored primers and quantified using LightCycler® 480 SYBR Green I Master in LightCycler® 480 Instrument II system (Roche) and analyzed by LightCycler® 480 Software 1.5.0.

### Cell culture and membrane preparation

CHO cells were grown to confluence in 75 cm^2^ flasks in Dulbecco’s modified Eagle’s medium (DMEM) supplemented with 10% fetal bovine serum. Two million cells were subcultured in 100-mm Petri dishes. The medium was supplemented with 5 mM sodium butyrate for the last 24 hours of cultivation to increase receptor expression. Cells were washed with phosphate-buffered saline and manually harvested on day 5 after subculture and centrifuged for 3 min at 250 × g. The pellet was suspended in 10 ml of ice-cold homogenization medium (100 mM NaCl, 20 mM Na-HEPES, 10 mM EDTA, pH = 7.4) and homogenized on ice by two 30 sec strokes using a Polytron homogenizer (Ultra-Turrax; Janke & Kunkel GmbH & Co. KG, IKA-Labortechnik, Staufen, Germany) with a 30-sec pause between strokes. Cell homogenates were centrifuged for 5 min at 1000 × g. The supernatant was collected and centrifuged for 30 min at 30,000 × g. Pellets were suspended in the washing medium (100 mM, 10 mM MgCl_2_, 20 mM Na-HEPES, pH = 7.4), left for 30 min at 4 °C, and then centrifuged again for 30 min at 30,000 × g. Resulting membrane pellets were kept at −80 °C until assayed.

### Radioligand binding experiments

All radioligand binding experiments were optimized and carried out according to general guidelines^23^. Membranes (5 to 10 μg of membrane proteins per sample for [^35^S]GTPγS binding or 20 to 50 μg of membrane proteins per sample for [^3^H]NMS binding) were used. Agonist binding was determined in competition experiments with 1 nM [^3^H]NMS. Membranes were incubated for 3 hours at 37 °C in 400 μl of Krebs-HEPES buffer (KHB; final concentrations in mM: NaCl 138; KCl 4; CaCl_2_ 1.3; MgCl_2_ 1; NaH_2_PO_4_ 1.2; HEPES 20; glucose 10; pH adjusted to 7.4). In saturation experiments of binding of [^3^H]N-methylscopolamine ([^3^H]NMS) six concentrations of the radioligand (ranging from 63 to 2000 pM) were used and incubation volume was increased to 0.8 ml. Nonspecific binding was determined in the presence of 10 μM atropine.

Agonist-stimulated [^35^S]GTPγS binding was measured in a final volume of 200 µl of washing medium with 500 pM [^35^S]GTPγS and 50 µM GDP for 20 min at 30 °C after 60 min preincubation with GDP and agonist. Nonspecific binding was determined in the presence of 1 µM unlabeled GTPγS.

Incubation was terminated by filtration through Whatman GF/C glass fiber filters (Whatman) using a Brandel harvester (Brandel, USA). Filters were dried in a microwave oven (3 min, 800 W) and then solid scintillator Meltilex A was melted on filters (90 °C, 70 s) using a hot plate. The filters were cooled and counted in a Wallac Microbeta scintillation counter (Wallac, Finland).

### Accumulation of Inositol phosphates

Accumulation of inositol phosphates (IP_X_) was assayed in cells grown in 96-well plates. The cells were loaded with 200 nM [^3^H]myo-inositol (ARC, USA) in DMEM overnight. Then DMEM was removed and the cells were washed with 100 µl KHB. Then cells were incubated with agonists at 200 µl of KHB containing 10 mM LiCl for 1 hour at 37 °C. Incubation was terminated by the removal of incubation medium and addition of 50 µl of 20% trichloracetic acid (TCA). Plates were kept at 4 °C for 1 hour, then 40 µl of TCA extract were transferred to another 96-well plate, mixed with 200 µl of Rotiszint scintillation cocktail and counted in Wallac Microbeta. Rest of TCA extract was discarded, individual wells were washed with 50 µl of 20% TCA, 50 µl of 1 M NaOH was added to each well and plates were shaken at room temperature for 15 min. Then 40 µl of NaOH lysate were transferred to another 96-well plate, mixed with 200 µl of Rotiszint scintillation cocktail (Carl Roth, Germany) and counted in Wallac Microbeta. Level of inositol phosphates was calculated as a fraction of soluble (TCA extract) to total (TCA extract plus NaOH lysate) radioactivity.

### Microfluorometry of free intracellular calcium

Cells grown on glass coverslips were washed twice with KHB and then prelabelled with 5 µM Fura 2-AM in KHB enriched with 1 mM pluronic P68 for one hour at 37 °C. After prelabelling cells were washed twice with KHB, mounted to a superfusion chamber, placed on a stage of Olympus IX-90 inverted fluorescent microscope, and continuously superfused at a flow rate 0.5 ml/min. Microfluorometry was set-up to measure kinetics of the functional response to agonists. Cells expressing fusion proteins of muscarinic receptors and G_15_ G-protein were exposed to 5 increrasing concentrations of agonist for 10 s. Individual exposures were separated by 10 min superfusion with agonist-free KHB medium. Images were recorded using a CCD camera connected to a computer equipped with Metafluor 7.0 software (Visitron Systems GmBH, Germany) for image acquisition and analysis. Images of the whole measured field containing about 40 cells were saved and analysed off-line after the measurements. Two pairs of images per second were recorded. Only responding cells were selected (by exclusion of weakly and/or slow responding cells or cells with abnormal response; the outliers in peak value, time to peak or fall time were identified by interquartile range (IQR) where data below Q1-1.5*IQR and above Q3 + 1.5*IQR were considered outliers) for further analysis. Calcium signals of selected cells were averaged, normalized to basal calcium level and further analysed by means of array-oriented program Grace (http://plasma-gate.weizmann.ac.il/Grace).

### Generation and analysis of theoretical data

Whole processes of the theoretical data generation, non-linear regression and analysis of the results were automated by scripts written in Python 2.7. Theoretical data with 3% of proportional noise were simulated by NumPy package. Non-linear regression was performed using optimize function of SciPy package. Standard deviations of estimated parameters were calculated from Jacobian covariance matrix. For control the standard deviations were computed by bootstrap method. Data and fits were visualized by Matplotlib package.

### Analysis of experimental data

Data from experiments were processed in Libre Office and then analyzed and plotted using program Grace (http://plasma-gate.weizmann.ac.il/Grace). Statistical analysis was performed using statistical package R (http://www.R-project.org). The following equations were used for non-linear regression analysis:

*[*^*3*^*H]NMS saturation binding*.

7 $$y=\frac{{B}_{{\rm{MAX}}}\ast x}{{\rm{x}}+{{\rm{K}}}_{D}}$$

where y is specific binding at free concentration x, B_MAX_ is the maximum binding capacity, and K_D_ is the equilibrium dissociation constant.

*Competition binding*.

8 $$y={\rm{100}}-\frac{({\rm{100}}-{f}_{{\rm{low}}})\ast x}{{\rm{x}}+{{\rm{IC}}}_{{\rm{50high}}}}-\frac{{f}_{{\rm{low}}}\ast x}{{\rm{x}}+{{\rm{IC}}}_{{\rm{50low}}}}$$

where y is specific radioligand binding at concentration x of competitor expressed as per cent of binding in the absence of competitor, IC_50_ is the concentration causing 50% inhibition of radioligand binding at high (IC_50high_) and low (IC_50low_) affinity binding sites, f_low_ is the fraction of low affinity binding sites expressed in per cent. Inhibition constant K_I_ was calculated as:

9 $${K}_{I}=\frac{{{\rm{IC}}}_{50}}{1+\frac{[D]}{{K}_{D}}}$$

where [D] is the concentration of radioligand used and K_D_ is its equilibrium dissociation constant.

*Concentration response curve*.

10 $$y=1+\frac{({{\rm{E}}^{\prime} }_{{\rm{MAX}}}-1)\ast {x}^{{\rm{nH}}}}{{x}^{{\rm{nH}}}+{{\rm{EC}}}_{{\rm{50}}}^{{\rm{nH}}}}$$

where y is response normalized to basal activity at concentration x, E’_MAX_ is apparent maximal response, EC_50_ is concentration causing half-maximal effect, and nH is Hill coefficient.

## Supplementary information

Supplementary information - text

Supplementary information - Python code
