# Supplementary material for: Applications and limitations of fitting of the operational model to determine relative efficacies of agonists
Source: Sci Rep. 2019 Mar 15;9:4637. doi: 10.1038/s41598-019-40993-w (PMC6420642; doi:10.1038/s41598-019-40993-w)
Supplement: Supplementary file 1 — Supplementary information - text [file 41598_2019_40993_MOESM1_ESM.pdf]

Supplementary information: Applications and limitations of fitting of the operational model to determine relative efficacies of agonists

Jan Jakubík[1,*], Alena Randáková[1], Vladimír Rudajev[1], Pavel Zimčík[1], Esam E. El-Fakahany[2], and Vladimír Doležal[1],

[1], Institute of Physiology CAS, 142 20 Prague, Czech Republic.

[2], Department of Experimental and Clinical Pharmacology, University of Minnesota College of Pharmacy, Minneapolis, MN 55455, USA.

[*]Correspondence to Jan Jakubík, Institute of Physiology CAS, Vídeňská 1083,142 20 Praha, Czech Republic, e-mail: jan.jakubik@fgu.cas.cz, phone: +420-2-4106-2620

Sequences receptor G-protein fusion proteins

M_2_G_{15}

MNNSTNSSNNSLALTSPYKTFEVVFIVLVAGSLSLVTIIGNILVMVSIKVNRHLQTVNNY
FLFSLACADLIIGVFSMNLYTLYTVIGYWPLGPVVCDLWLALDYVVSNASVMNLLIISFD
RYFCVTKPLTYPVKRTTKMAGMMIAAAWVLSFILWAPAILFWQFIVGVRTVEDGECYIQF
FSNAAVTFGTAIAAFYLPVIIMTVLYWHISRASKSRIKKDKKEPVANQDPVSPSLVQGRI
VKPNNNNMPSSDDGLEHNKIQNGKAPRDPVTENCVQGEEKESSNDSTSVSAVASNMRDDE
ITQDENTVSTSLGHSKDENSKQTCIRIGTKTPKSDSCTPTNTTVEVVGSSGQNGDEKQNI
VARKIVKMTKQPAKKKPPPSREKKVTRTILAILLAFIITWAPYNVMVLINTFCAPCIPNT
VWTIGYWLCYINSTINPACYALCNATFKKTFKHLLMCHYKNTGATRARSLKWRCCPWCLT
EDEKAAARVDQEINRILLEQKKQDRGELKLLLLGPGESGKSTFIKQMRIIHGAGYSEEER
KGFRPLVYQNIFVSMRAMIEAMERLQIPFSRPESKHHASLVMSQDPYKVTTFEKRYAAAM
QWLWRDAGIRACYERRREFHLLDSAVYYLSHLERITEEGYVPTAQDVLRSRMPTTGINEY
CFSVQKTNLRIVDVGGQKSERKKWIHCFENVIALIYLASLSEYDQCLEENNQENRMKESL
ALFGTILELPWFKSTSVILFLNKTDILEEKIPTSHLATYFPSFQGPKQDAEAAKRFILDM
YTRMYTGCVDGPEGSKKGARSRRLFSHYTCATDTQNIRKVFKDVRDSVLARYLDEINLL*

M_4_G_{15}

MANFTPVNGSSGNQSVRLVTSSSHNRYETVEMVFIATVTGSLSLVTVVGNILVMLSIKVN
RQLQTVNNYFLFSLACADLIIGAFSMNLYTVYIIKGYWPLGAVVCDLWLALDYVVSNASV
MNLLIISFDRYFCVTKPLTYPARRTTKMAGLMIAAAWVLSFVLWAPAILFWQFVVGKRTV
PDNQCFIQFLSNPAVTFGTAIAAFYLPVVIMTVLYIHISLASRSRVHKHRPEGPKEKKAK
TLAFLKSPLMKQSVKKPPPGEAAREELRNGKLEEAPPPALPPPPRPVADKDTSNESSSGS
ATQNTKERPATELSTTEATTPAMPAPPLQPRALNPASRWSKIQIVTKQTGNECVTAIEIV
PATPAGMRPAANVARKFASIARNQVRKKRQMAARERKVTRTIFAILLAFILTWTPYNVMV
LVNTFCQSCIPDTVWSIGYWLCYVNSTINPACYALCNATFKKTFRHLLLCQYRNIGTTGA
TRARSLKWRCCPWCLTEDEKAAARVDQEINRILLEQKKQDRGELKLLLLGPGESGKSTFI
KQMRIIHGAGYSEEERKGFRPLVYQNIFVSMRAMIEAMERLQIPFSRPESKHHASLVMSQ
DPYKVTTFEKRYAAAMQWLWRDAGIRACYERRREFHLLDSAVYYLSHLERITEEGYVPTA
QDVLRSRMPTTGINEYCFSVQKTNLRIVDVGGQKSERKKWIHCFENVIALIYLASLSEYD
QCLEENNQENRMKESLALFGTILELPWFKSTSVILFLNKTDILEEKIPTSHLATYFPSFQ
GPKQDAEAAKRFILDMYTRMYTGCVDGPEGSKKGARSRRLFSHYTCATDTQNIRKVFKDV
RDSVLARYLDEINLL*

Procedure of simulation and fitting of theoretical datasets

Theoretical concentration-response curves (CRC) were simulated using Pyhton script OM_simulate_data.py.

For model analysis 1 set (5 CRCs Data_A.dat … Data_E.dat) was generated. Tau values were set to 0.1, 1, 10, 100 and 1000 respectively. LogK_A value was set to -6 and E_{MAX} value to 1. Proportion of random noise was set to ±3%. Then OM was fitted to CRCs either directly using script OM_fit_5.py or by two-step procedure using script FR_to_OM.py. OM_fit_5.py first fits Eq. a to individual CRCs, then fits Eq. a to all 5 CRCs with shared E_{MAX} and finally fits Eq. a to all 5 CRCs with shared E_{MAX} and K_A. FR_to_OM.py first fits Eq. 4 to individual CRCs, then fits Eq. f to resulting EC_{50} and E'_{MAX} values and finally fits Eq. a to individual CRCs with E_{MAX} and K_A fixed to values calculated in previous step using Eq. f.

For analysis of τ estimation distribution 1000 sets of CRCs were generated using cript OM_simulate_data.py. Then Eq. a was fitted to all sets using OM_fit_tau.py. In parallel, Eq. a with τ in form of logarithm was fitted to all sets using OM_fit_logtau.py. Distribution of estimated τ values was analysed using OM_analyze_tau_distribution.py and OM_analyze_logtau_distribution.py, respectively.

Figure SI1 Fitting OM to simulated data

Simulation parameters log K_A = -6, E_{MAX} = 1; curve A, τ = 0.1; curve B, τ = 1; curve C, τ = 10; curve D, τ = 100; curve B, τ = 1000. Black – optimal initial estimates; red – underestimated E_{MAX}; blue – overestimated E_{MAX}. Values are parameter estimates ± SD.

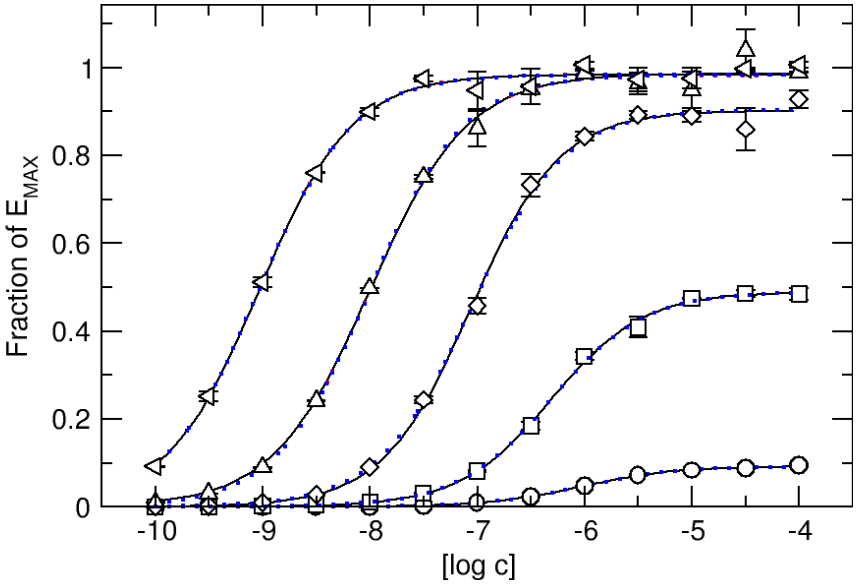

Optimal initial estimates			
Dataset	τ	E_{MAX}	log K_A
A	0.1017 ± 143155	1.00 ± 1278802	-5.98 ± 56434
B	0.9770 ± 963950	0.99 ± 494776	-6.01 ± 211757
C	10.28 ± 8530181	0.99 ± 73080	-6.00 ± 328416
D	99.43 ± 109608398	0.99 ± 10893	-6.00 ± 473965
E	1074 ± 558660685	0.98 ± 476	-6.00 ± 225560
Underestimated E_{MAX} (initial estimate 0.5)			
Dataset	τ	E_{MAX}	log K_A
A (τ_{ini} = 0.5)	0.2868 ± 534965	0.41 ± 600526	-5.91 ± 180556
B (τ_{ini} = 5)	2.199 ± 2647699	0.71 ± 268141	-5.80 ± 359401
C (τ_{ini} = 50)	14.89 ± 14779923	0.97 ± 60323	-5.85 ± 403846
D (τ_{ini} = 500)	176.7 ± 180782233	0.99 ± 5688	-5.75 ± 441795
E (τ_{ini} = 5000)	2678 ± 1417584128	0.98 ± 194	-5.60 ± 229731
Overestimated E_{MAX} (initial estimate 5)			
Dataset	τ	E_{MAX}	log K_A
A (τ_{ini} = 0.05)	0.0319 ± 73163	2.99 ± 6646720	-6.01 ± 30793
B (τ_{ini} = 0.5)	0.4162 ± 544165	1.67 ± 1538812	-6.16 ± 166871
C (τ_{ini} = 5)	5.545 ± 4624230	1.07 ± 136137	-6.24 ± 306809
D (τ_{ini} = 50)	139.5 ± 109208206	0.99 ± 5512	-5.85 ± 337511
E (τ_{ini} = 500)	1811 ± 1114552613	0.98 ± 334	-5.77 ± 267028

Figure SI2 Distribution of estimated τ and logτ values

1000 sets of CRCs was generated and then distribution histograms of τ and logτ values were plotted.

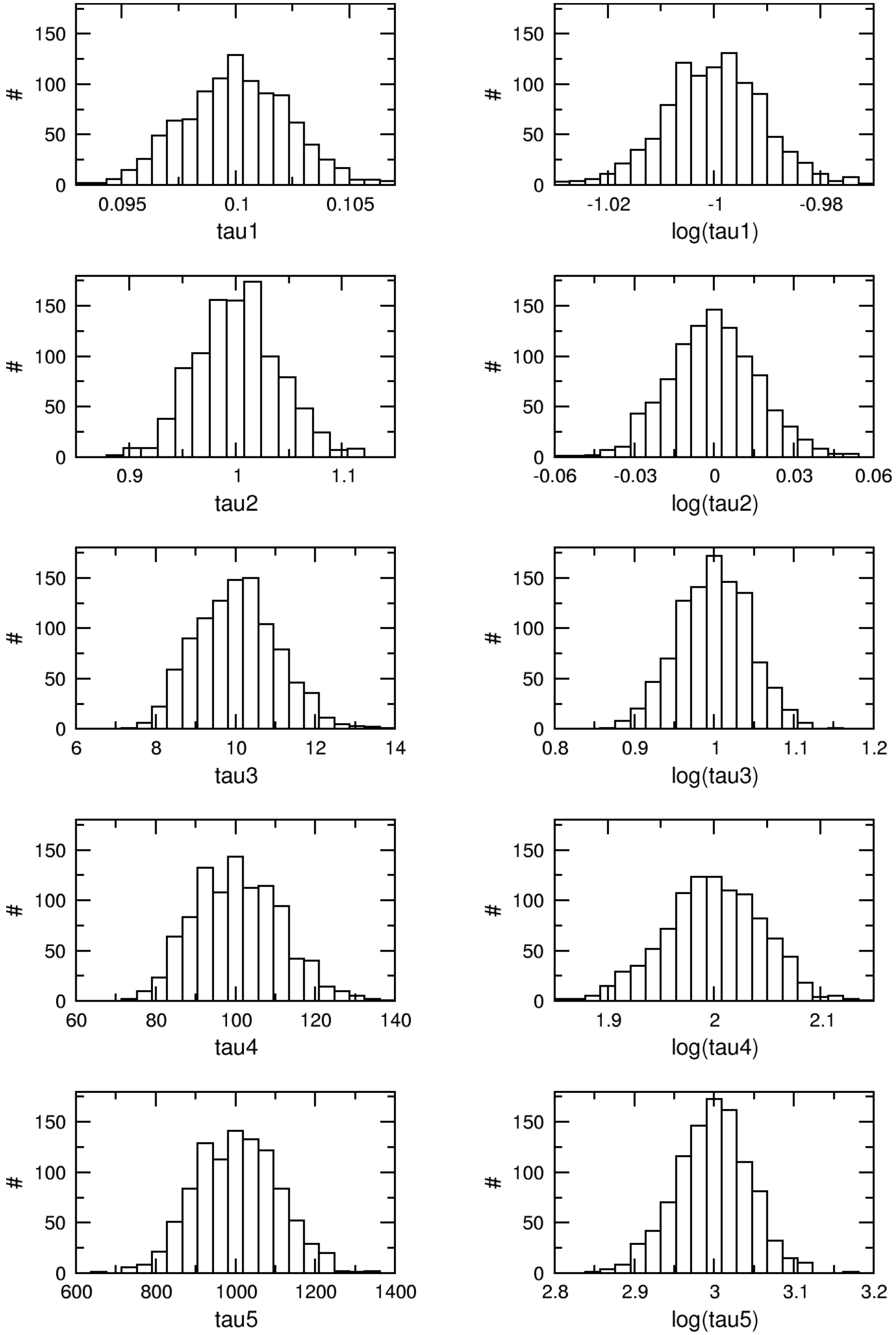

Figure SI3 Functional response to carbachol at $M_2\_G_{15}$

Curves and parameters were obtained by fitting Eq. a to the functional response data with K_A fixed to value of K_I from binding experiments (Main manuscript, Table 4).

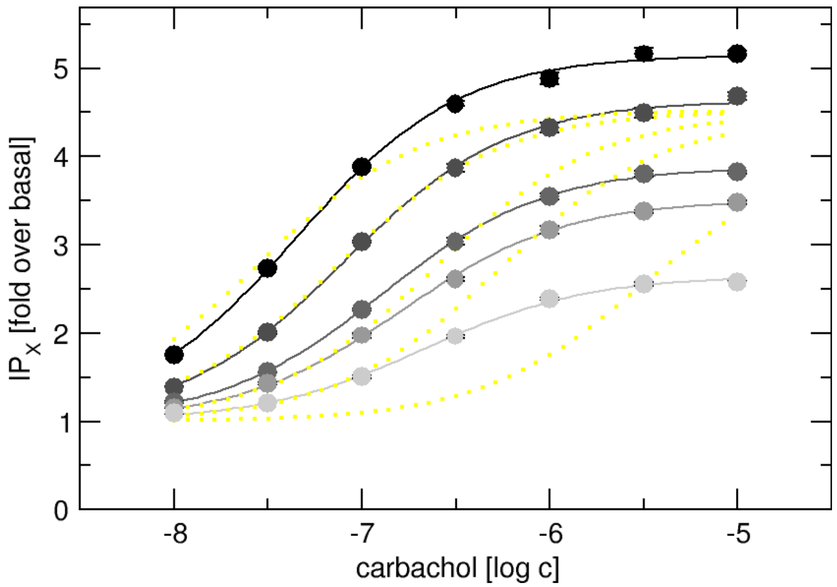

Individual fits			
	τ	E_{MAX}	log K_A
$M_2\_G_{15}$ #1	523 ± 22	5.16 ± 0.03	-4.63
$M_2\_G_{15}$ #2	300 ± 13	4.65 ± 0.03	-4.61
$M_2\_G_{15}$ #3	177 ± 4	3.90 ± 0.01	-4.64
$M_2\_G_{15}$ #4	142 ± 5	3.52 ± 0.02	-4.63
$M_2\_G_{15}$ #5	103 ± 5	2.66 ± 0.02	-4.64
Global fit with shared E_{MAX}			
	τ	E_{MAX}	log K_A
$M_2\_G_{15}$ #1	844 ± 742	4.53 ± 0.35	-4.63
$M_2\_G_{15}$ #2	316 ± 262	4.53 ± 0.35	-4.61
$M_2\_G_{15}$ #3	94 ± 78	4.53 ± 0.35	-4.64
$M_2\_G_{15}$ #4	42 ± 33	4.53 ± 0.35	-4.63
$M_2\_G_{15}$ #5	6.6 ± 5.1	4.53 ± 0.35	-4.64

Figure SI4 Functional response to oxotremorine at M_2_G_{15}

Curves and parameters were obtained by fitting Eq. a to the functional response data with K_A fixed to value of K_I from binding experiments (Main manuscript, Table 4).

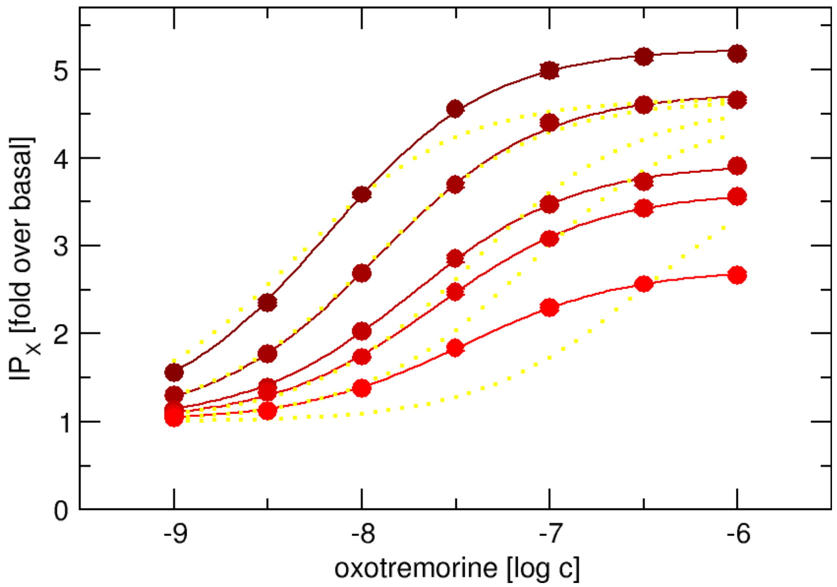

Individual fits			
	τ	E_{MAX}	log K_A
M_2_G_{15} #1	290 ± 6	5.26 ± 0.02	-5.72
M_2_G_{15} #2	142 ± 4	4.76 ± 0.02	-5.76
M_2_G_{15} #3	87 ± 3	3.96 ± 0.02	-5.78
M_2_G_{15} #4	71 ± 2	3.65 ± 0.02	-5.75
M_2_G_{15} #5	53 ± 1	2.76 ± 0.01	-5.73
Global fit with shared E_{MAX}			
	τ	E_{MAX}	log K_A
M_2_G_{15} #1	409 ± 282	4.68 ± 0.38	-5.72
M_2_G_{15} #2	156 ± 101	4.68 ± 0.38	-5.76
M_2_G_{15} #3	45 ± 30	4.68 ± 0.38	-5.78
M_2_G_{15} #4	23 ± 14	4.68 ± 0.38	-5.75
M_2_G_{15} #5	4.6 ± 2.8	4.68 ± 0.38	-5.73

Figure SI5 Functional response to pilocarpine at M_2_G_{15}

Curves and parameters were obtained by fitting Eq. a to the functional response data with K_A fixed to value of K_I from binding experiments (Main manuscript, Table 4).

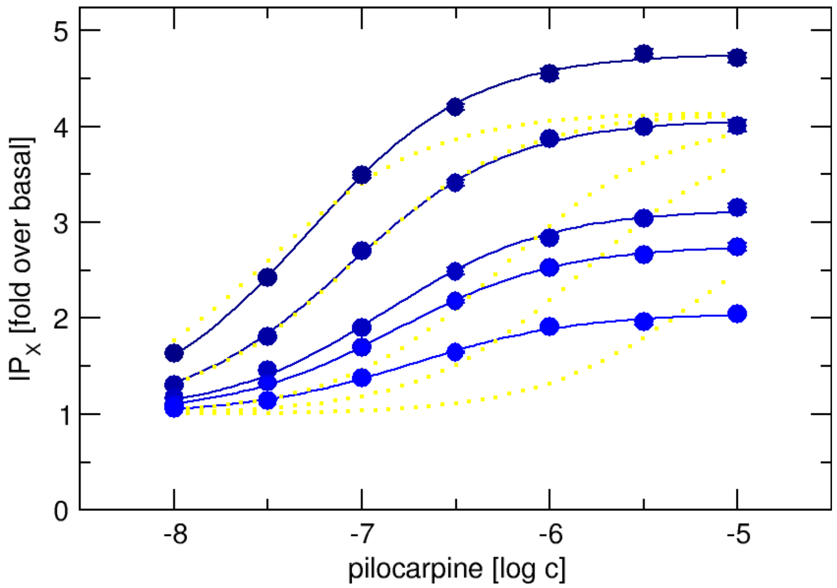

Individual fits			
	τ	E_{MAX}	log K_A
M_2_G_{15} #1	629 ± 19	4.76 ± 0.02	-4.49
M_2_G_{15} #2	358 ± 14	4.08 ± 0.02	-4.52
M_2_G_{15} #3	229 ± 13	3.14 ± 0.03	-4.51
M_2_G_{15} #4	189 ± 5	2.77 ± 0.01	-4.54
M_2_G_{15} #5	168 ± 12	2.06 ± 0.02	-4.50
Global fit with shared E_{MAX}			
	τ	E_{MAX}	log K_A
M_2_G_{15} #1	1011 ± 984	4.15 ± 0.38	-4.49
M_2_G_{15} #2	359 ± 346	4.15 ± 0.38	-4.52
M_2_G_{15} #3	52 ± 48	4.15 ± 0.38	-4.51
M_2_G_{15} #4	21 ± 19	4.15 ± 0.38	-4.54
M_2_G_{15} #5	3.9 ± 3.8	4.15 ± 0.38	-4.50

Table SI6 Functional response to carbachol at $M_4\_G_{15}$

Curves and parameters were obtained by fitting Eq. a to the functional response data with K_A fixed to value of K_I from binding experiments (Main manuscript, Table 4).

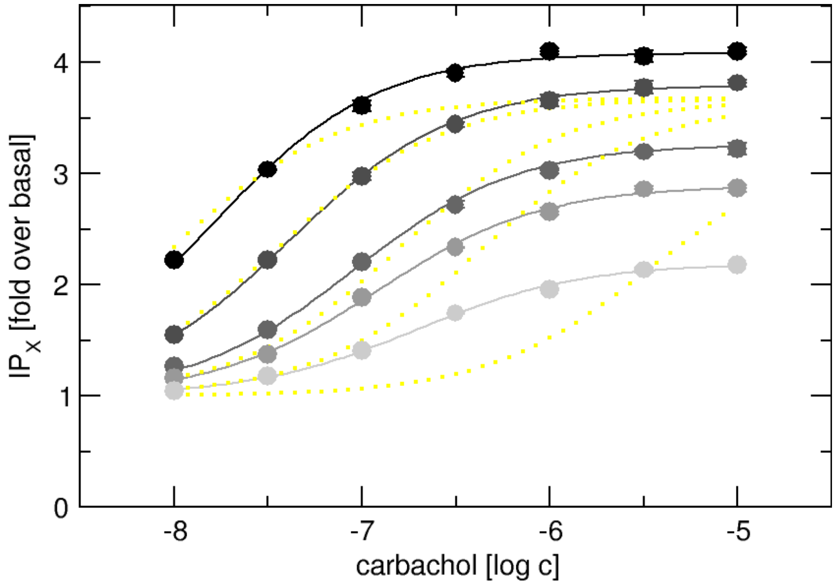

Individual fits			
	τ	E_{MAX}	log K_A
$M_4\_G_{15}$ #1	2910 ± 166	4.18 ± 0.09	-4.63
$M_4\_G_{15}$ #2	539 ± 14	3.80 ± 0.01	-4.65
$M_4\_G_{15}$ #3	276 ± 11	3.23 ± 0.02	-4.62
$M_4\_G_{15}$ #4	191 ± 10	2.90 ± 0.02	-4.63
$M_4\_G_{15}$ #5	120 ± 7	2.20 ± 0.02	-4.62
Global fit with shared E_{MAX}			
	τ	E_{MAX}	log K_A
$M_4\_G_{15}$ #1	2341 ± 2295	3.68 ± 0.31	-4.63
$M_4\_G_{15}$ #2	689 ± 553	3.68 ± 0.31	-4.65
$M_4\_G_{15}$ #3	151 ± 91	3.68 ± 0.31	-4.62
$M_4\_G_{15}$ #4	55 ± 45	3.68 ± 0.31	-4.63
$M_4\_G_{15}$ #5	6.0 ± 2.5	3.68 ± 0.31	-4.62

Figure SI7 Functional response to oxotremorine at $M_4\_G_{15}$

Curves and parameters were obtained by fitting Eq. a to the functional response data with K_A fixed to value of K_I from binding experiments (Main manuscript, Table 4).

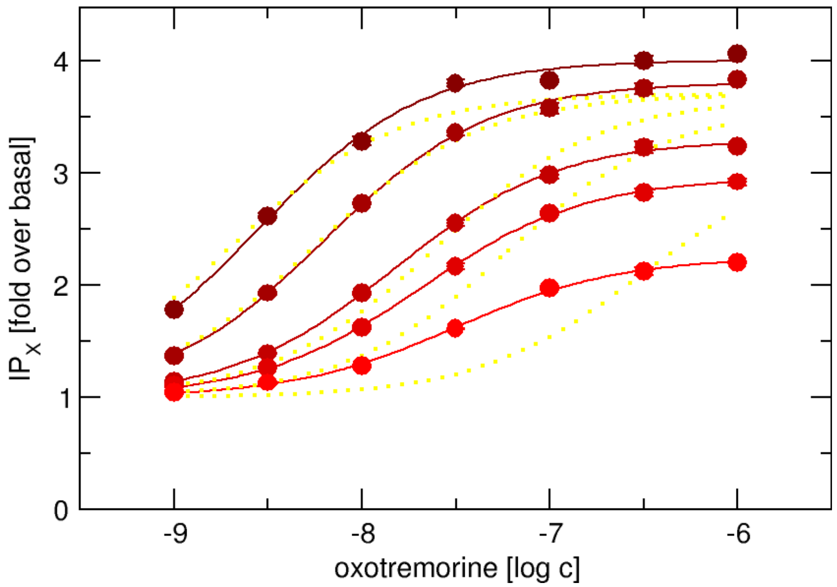

Individual fits			
	τ	E_{MAX}	log K_A
$M_4\_G_{15}$ #1	484 ± 34	4.01 ± 0.04	-5.86
$M_4\_G_{15}$ #2	225 ± 9	3.82 ± 0.02	-5.84
$M_4\_G_{15}$ #3	98 ± 3	3.32 ± 0.02	-5.83
$M_4\_G_{15}$ #4	75 ± 2	2.99 ± 0.01	-5.80
$M_4\_G_{15}$ #5	46 ± 2	2.28 ± 0.02	-5.82
Global fit with shared E_{MAX}			
	τ	E_{MAX}	log K_A
$M_4\_G_{15}$ #1	798 ± 382	3.71 ± 0.34	-5.86
$M_4\_G_{15}$ #2	275 ± 156	3.71 ± 0.34	-5.84
$M_4\_G_{15}$ #3	61 ± 22	3.71 ± 0.34	-5.83
$M_4\_G_{15}$ #4	24 ± 8	3.71 ± 0.34	-5.80
$M_4\_G_{15}$ #5	4.0 ± 1.5	3.71 ± 0.34	-5.82

Figure SI8 Functional response to pilocarpine at $M_4\_G_{15}$

Curves and parameters were obtained by fitting Eq. a to the functional response data with K_A fixed to value of K_I from binding experiments (Main manuscript, Table 4).

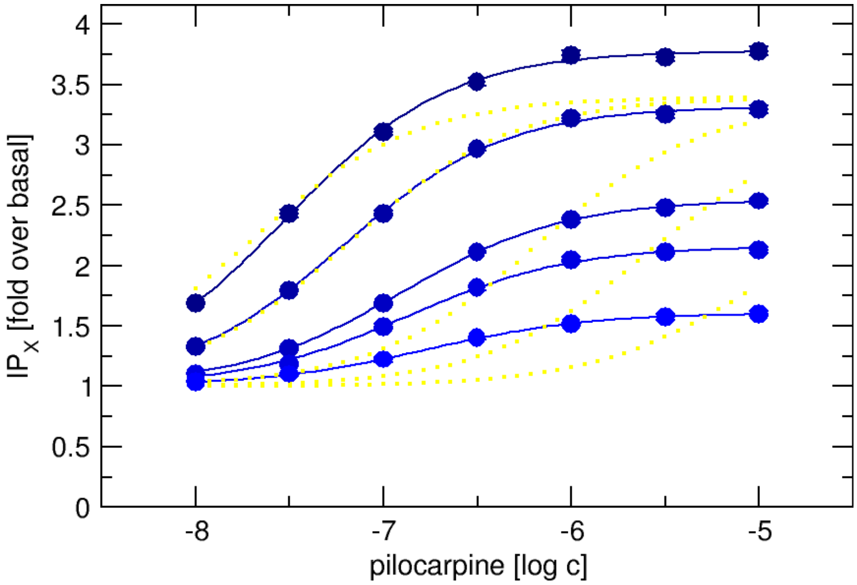

Individual fits			
	τ	E_{MAX}	log K_A
$M_4\_G_{15}$ #1	921 ± 28	3.78 ± 0.01	-4.55
$M_4\_G_{15}$ #2	475 ± 14	3.32 ± 0.01	-4.54
$M_4\_G_{15}$ #3	247 ± 4	2.55 ± 0.01	-4.51
$M_4\_G_{15}$ #4	218 ± 15	2.17 ± 0.02	-4.53
$M_4\_G_{15}$ #5	176 ± 5	1.61 ± 0.00	-4.52
Global fit with shared E_{MAX}			
	τ	E_{MAX}	log K_A
$M_4\_G_{15}$ #1	1927 ± 1504	3.40 ± 0.37	-4.55
$M_4\_G_{15}$ #2	533 ± 386	3.40 ± 0.37	-4.54
$M_4\_G_{15}$ #3	51 ± 38	3.40 ± 0.37	-4.51
$M_4\_G_{15}$ #4	12 ± 9	3.40 ± 0.37	-4.53
$M_4\_G_{15}$ #5	3.3 ± 2.4	3.40 ± 0.37	-4.52
